# One-year infection control rates of a DAIR (debridement, antibiotics and implant retention) procedure after primary and prosthetic-joint-infection-related revision arthroplasty – a retrospective cohort study

**DOI:** 10.5194/jbji-6-91-2021

**Published:** 2021-01-27

**Authors:** F. Ruben H. A. Nurmohamed, Bruce van Dijk, Ewout S. Veltman, Marrit Hoekstra, Rob J. Rentenaar, Harrie H. Weinans, H. Charles Vogely, Bart C. H. van der Wal

**Affiliations:** 1 Department of Orthopedics, University Medical Center Utrecht, Utrecht, the Netherlands; 2 Department of Microbiology, University Medical Center Utrecht, Utrecht, the Netherlands; 3 Department of BioMechanical Engineering, Delft University of Technology, Delft, the Netherlands

## Abstract

**Introduction**: Debridement, antibiotics and implant retention (DAIR) procedures are effective treatments for acute postoperative or acute hematogenous periprosthetic joint infections. However, literature reporting on the effectiveness of DAIR procedures performed after a one- or two-stage revision because of a prosthetic joint infection (PJI) (PJI-related revision arthroplasty) is scarce. The aim of this study is to retrospectively evaluate the infection control after 1 year of a DAIR procedure in the case of an early postoperative infection either after primary arthroplasty or after PJI-related revision arthroplasty. **Materials and methods**: All patients treated with a DAIR procedure within 3 months after onset of PJI between 2009 and 2017 were retrospectively included. Data were collected on patient and infection characteristics. All infections were confirmed by applying the Musculoskeletal Infection Society (MSIS) 2014 criteria. The primary outcome was successful control of infection at 1 year after a DAIR procedure, which was defined as the absence of clinical signs, such as pain, swelling, and erythema; radiological signs, such as protheses loosening; or laboratory signs, such as C-reactive protein (CRP) (<10) with no use of antibiotic therapy. **Results**: Sixty-seven patients were treated with a DAIR procedure (41 hips and 26 knees). Successful infection control rates of a DAIR procedure after primary arthroplasty (n=51) and after prior PJI-related revision arthroplasty (n=16) were 69 % and 56 %, respectively (p=0.38). The successful infection control rates of a DAIR procedure after an early acute infection (n=35) and after a hematogenous infection (n=16) following primary arthroplasty were both 69 % (p=1.00). **Conclusion**: In this limited study population, no statistically significant difference is found in infection control after 1 year between DAIR procedures after primary arthroplasty and PJI-related revision arthroplasty.

27 January 2021

## 1 Introduction

Prosthetic joint infection (PJI) is a devastating complication following total hip and knee arthroplasty. The incidences of PJI in the Western countries are reported to range up to 4 % for primary total hip and knee arthroplasty and even as high as 20 % following revision arthroplasty (Ahmed et al., 2020; Hosny and Keenan, 2020).

The most common clinical signs of acute and hematogenous PJI include acute pain, erythema and fever (Barrett and Atkins, 2014; Zimmerli, 2006). A debridement, antibiotics and implant retention (DAIR) procedure is the treatment of choice for acute PJI of the hip and knee (Chotanaphuti et al., 2019; Sukeik and Haddad, 2019; Wouthuyzen-Bakker et al., 2020). A recent systematic review and meta-analysis reported a wide range of success percentages for DAIR procedures from 11 % to 100 % (Kunutsor et al., 2018; Tsang et al., 2017). Several studies have shown that the time between onset of symptoms and the DAIR procedure is strongly associated with the success rate of treatment (Karczewski et al., 2019; Kuiper et al., 2013; Kunutsor et al., 2018; Sendi et al., 2017). Nonetheless, even more than 6 weeks after the index arthroplasty an eradication rate of 60 % can be achieved when performing DAIR (Löwik et al., 2019). The success rate of DAIR for early postoperative infection is better than for hematogenous infections (Volpin et al., 2016; Wouthuyzen-Bakker et al., 2020). There is no place for DAIR in the treatment of chronic infections (Sukeik and Haddad, 2019). Prior infection in another prosthetic joint and prior two-stage exchange for PJI of the same joint are both reported to worsen the infection eradication rate of a repeated revision procedure compared to a first revision (Chalmers et al., 2019; Khan et al., 2019). However, the effectiveness of DAIR procedures after prior PJI-related revision arthroplasty is still up for debate.

The primary aim of this study is to retrospectively evaluate the infection control rate of DAIR procedures performed after a one- or two-stage revision because of a PJI (PJI-related revision arthroplasty) in comparison to DAIR procedures performed after primary arthroplasty. The secondary aim of this study is to evaluate if the infection control rate of a DAIR procedure after primary arthroplasty depends on whether an infection is early postoperative or hematogenous. We hypothesize that previous PJI-related revision arthroplasty procedures have a negative effect on the infection control rate of subsequently performed DAIR procedures.

## 2 Methods

In this observational study, we reviewed the records of all patients in our prospectively collected database who had an infection treatment of the hip or knee in our hospital between 2009 and 2017. After approval, we reviewed the records of all patients in our prospectively collected database who had an infection treatment of the hip or knee in our hospital between 2009 and 2017. We included all patients with one periprosthetic joint infection. All DAIR procedures were performed after placing a primary hip or knee prothesis or after full reimplantation of a hip or knee prothesis for infection revision surgery. In all patients, diagnosis of infection was affirmed according to the Musculoskeletal Infection Society criteria (Parvizi and Gehrke, 2014). In our institution, DAIR procedures are only performed within 3 months after the onset of symptoms.

We retrieved general patient and infection characteristics, complications during treatment, and final outcomes from patients' records. Primary outcome was tier 1 infection control after DAIR treatment, based on the outcome-reporting tool suggested by the Musculoskeletal Infection Society workgroup (Fillingham et al., 2019). The absence of clinical signs, such as pain, swelling, and erythema; radiological signs, such as protheses loosening; or laboratory signs, such as CRP (<10), with no use of antibiotic therapy at the final follow-up 1 year after the first subsequently performed DAIR procedure was seen as a successful outcome. We used the presence of a prior PJI-related revision procedure and type of infection (acute early or hematogenous) as variables to separately analyze whether the infection control rate was affected (McPherson et al., 2002).

Failure of treatment was defined as failed control of the periprosthetic infection. This includes tier 2 or higher based on the aforementioned outcome-reporting tool. Specifically, additional surgeries, such as resection arthroplasty, arthrodesis or amputation of the limb, or the administration of suppressive antibiotics prior to the final 1-year postoperative follow-up was seen as a failed outcome. Only a third repeated DAIR procedure was considered failure of treatment.

### 2.1 Patient characteristics

Characteristics such as age, sex, BMI (body mass index), ASA class (American Society of Anesthesiologists), smoking or alcohol use, co-morbidities, and previous infection treatment were extracted from the medical charts. Previous PJI-related procedures were subdivided into (multiple) DAIR procedures and (one- or two-stage) revision procedures.

### 2.2 Prosthetic joint infection characteristics

PJI characteristics included location of infection, type of infection and involvement of soft tissue. To determine the degree of compromise of the host and the infection site, the McPherson staging system was used (McPherson et al., 2002). For early acute infections, defined as onset of infection within 3 months after surgery, the time between primary or PJI-related revision arthroplasty and the DAIR procedure was used as the infection period. For acute hematogenous infections, defined as infections spread from a distant infectious focus, the time from onset of symptoms until the DAIR procedure was calculated as the infection period.

### 2.3 The DAIR procedure

A debridement, antibiotics and implant retention procedure consists of several steps in consecutive order. First, six synovial fluid or tissue samples are collected for culture and a meticulous debridement of the joint is performed to remove all infected tissue. The interchangeable parts (insert of the knee and inlay and head of the hip) are removed to facilitate posterior joint debridement and are then replaced. During surgery, the joint is extensively lavaged with 6 L of NaCl 0.9 %.

All patients were treated with cefazolin until culture results were available. When culture results were available, the definite antibiotic strategy was determined according to the found pathogen and antibiotic susceptibility test results in close consultation with a medical microbiologist and an infectious disease specialist. Patients generally received 3 months of antibiotic treatment following DAIR.

### 2.4 Statistical analysis

Descriptive statistics, mean and range are used to represent the demographics of the patients. Fisher's exact test was used to assess the level of significance for differences between the infection control rates of the groups. A P value <0.05 was considered to be statistically significant. Calculations and statistical analyses were performed using Excel and SPSS software (version 27).

**Figure 1:**
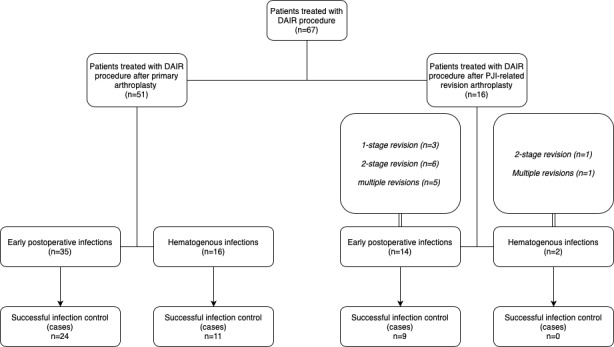
Flow diagram showing the infection characteristics, previous PJI procedures and number of successfully controlled infections.

**Figure 2:**
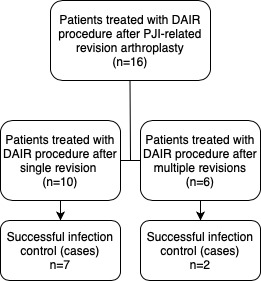
Flow diagram showing the numbers of patients with successfully controlled infections according to the frequency of previous PJI-related revision arthroplasty.

**Table 1:** Baseline patient characteristics.

	Total (n)	Successful infection control (n)	Failed infection control (n)
Number of patients	67	44 (66 %)	23 (34 %)
Hip	41	29 (71 %)	12 (29 %)
Knee	26	15 (58 %)	11 (42 %)
Mean age (range)	67 (18–92)	68 (18–92)	63 (35–78)
Gender M/F	29/38	16 (55 %)/28 (74 %)	13 (45 %)/10 (26 %)
Mean BMI (range)	27 (19–45)	27 (19–44)	28 (19–45)
Mean duration of infection (days)	20	22	16
Risk factors			
Smoking	12	7 (58 %)	5 (42 %)
Alcohol abuse	7	6 (86 %)	1 (14 %)
ASA 1/2/3	6/37/24	4/23/17	2/14/7
Host score (according to McPherson)			
Uncompromised	19	9 (47 %)	10 (53 %)
Compromised	44	32 (73 %)	12 (27 %)
Significantly compromised	4	3 (75 %)	1 (25 %)
Local extremity grade (according to McPherson)			
Uncompromised	58	41 (71 %)	17 (29 %)
Compromised	9	3 (33 %)	6 (67 %)

**Table 2:** Microbiology findings.

	Cases (n)	Successful infection control (n)
*Staphylococcus aureus*	13	8
*Staphylococcus epidermidis*	14	10
Other staphylococci${}^{a}$	5	5
Beta-hemolytic streptococci${}^{b}$	4	3
Enterococci${}^{c}$	6	2
Enterobacterales${}^{d}$	6	5
*Pseudomonas aeruginosa*	2	2
Other pathogens${}^{e}$	7	4
Polymicrobial	6	2
No organism identified	4	3
Total	67	44

${}^{a}$ *S. capitis* (n=2), *S. warneri* (n=1), *S. haemolyticus* (n=1) and *S. pseudintermedius* (n=1).${}^{b}$ *S. dysgalactiae* (n=3) and *S. agalactiae* (n=1).${}^{c}$ *Enterococcus faecalis* (n=3) and *E. faecium* (n=3).${}^{d}$ *Escherichia coli* (n=2), *Klebsiella pneumoniae* (n=2), *Enterobacter cloacae* complex (n=1) and *Serratia marcescens* (n=1).${}^{e}$ *Corynebacterium striatum* (n=1), *Corynebacterium tuberculostearicum* (n=1), *Anaerococcus hydrogenalis* (n=1), *Cutibacterium acnes* (n=3) and *Ureaplasma parvum* (n=1).

## 3 Results

Between 2009 and 2017, sixty-seven patients were treated for acute PJI with a DAIR procedure. Forty-one hip and 26 knee surgeries were performed. General patient and infection characteristics are shown in Figs. 1 and 2 and Tables 1 and 2. All patients had a follow-up 1 year after the first DAIR procedure. Overall, the infection was eradicated in 44 out of 67 patients.

### 3.1 DAIR procedure after primary arthroplasty versus after previous PJI-related revision arthroplasty

In 51 patients a DAIR procedure was performed after primary arthroplasty. In 16 patients a DAIR procedure was performed after a previous PJI-related revision procedure. A flow diagram of included patients and type of PJI-related revision arthroplasty can be found in Fig. 1. For the two patients with a hematogenous infection after previous PJI-related revision arthroplasty, the interval between revision surgery and the onset of hematogenous infection were 215 and 722 d. The infection control rate of DAIR procedures performed after primary arthroplasty was 69 % (35 out of 51 cases). For hip and knee cases, the infection control rate was 72 % (21 out of 29 cases) and 64 % (14 out of 22 cases), respectively. The infection control rate of DAIR procedures performed after PJI-related revision arthroplasty was 56 % (9 out of 16 cases). For hip and knee cases, the infection control rate was 67 % (8 out of 12 cases) and 25 % (1 out of 4 cases), respectively. There was no statistically significant difference in the infection control rate between DAIR procedures performed after primary arthroplasty and after PJI-related revision arthroplasty (p=0.38).

### 3.2 DAIR after early acute versus hematogenous infections after primary arthroplasty

In 35 patients a DAIR procedure was performed for an early acute infection following primary arthroplasty. In 16 patients a DAIR procedure was performed for an acute hematogenous infection (Fig. 1). The mean duration of symptoms was 12 d (0–83 d) for hematogenous infections. The infection control rate of DAIR procedures for early acute infections was 69 % (24 out of 35 cases). For hip and knee cases, infection control rate was 74 % (17 out of 23 cases) and 58 % (7 out of 12 cases), respectively. The infection control rate of DAIR procedures performed for hematogenous infections was 69 % (11 out of 16 cases). For hip and knee cases, the infection control rate was 67 % (4 out of 6 cases) and 70 % (7 out of 10 cases), respectively. There was no statistically significant difference in the infection control rate between these two groups (p=1.00).

### 3.3 Microbiology findings

The microbiology culture results of the tissue cultures taken during DAIR treatment can be found in Table 2. Sixty-three cases had positive perioperative findings. No pathogen was identified in four cases. In two of these cases, preoperative antibiotic treatment was administered by the referring physician prior to surgery.

## 4 Discussion

This study retrospectively evaluated the infection control rate of DAIR procedures performed for PJI after primary arthroplasty or after previous PJI-related revision arthroplasty of the hip and knee in a tertiary referral center. The infection control rate of DAIR procedures after primary arthroplasty was 69 % (35 out of 51 cases) compared to 56 % (9 out of 16 cases) for DAIR procedures after previous PJI-related revision arthroplasty. Our study population is too small to draw definite conclusions; however, these results show a trend that previous PJI treatment could have a negative effect on the infection control rate of DAIR procedures.

There seems to be a contrast between the infection control rates of DAIR performed after primary arthroplasty and DAIR after PJI-related revision arthroplasty. Even though the infection control rate for DAIR procedures after previous PJI-related revision arthroplasty is reduced, our data show that about 6 out of 10 infections can still be controlled without further major revision surgery. Furthermore, only 2 out of 6 DAIR procedures after multiple PJI-related revision arthroplasty procedures were successful, whereas the infection was controlled in 7 out of 10 DAIR procedures after a single PJI-related revision procedure (p=0.30) (Fig. 2). The infection control rate of DAIR treatment seems to further decline as the number of previously performed PJI-related revision arthroplasty procedures increases.

Most of our infection control rates of DAIR procedures are comparable to the overall pooled success rate of 61.4 % reported in a recent meta-analysis (Kunutsor et al., 2018). However, some other retrospective studies have reported higher infection control rates for DAIR procedures (Byren et al., 2009; Sendi et al., 2017; Volpin et al., 2016; Wouthuyzen-Bakker et al., 2020). The relatively large number of patients with ASA 3 and McPherson compromised host score (Table 1) may cause a lower successful control rate in our population.

Literature on the results of DAIR procedures after revision surgery is scarce. Byren and colleagues reported a failure rate of 35 % and a 3.1 times increase in hazard ratio for failure of DAIR if it is performed after revision arthroplasty compared to after primary arthroplasty (Byren et al., 2009). Shohat and colleagues found no significant difference in the success rate if DAIR was performed after primary or revision arthroplasty (p=0.182) (Shohat et al., 2020). Lastly, Wouthuyzen-Bakker and colleagues found unadjusted and adjusted odds ratios of 1.65 (p=0.04) and 0.96 (p=0.90), respectively, for failure of a DAIR performed on revised prostheses with late prosthetic joint infections (Wouthuyzen-Bakker et al., 2019). Considering that no significant difference is found in the infection control rate of DAIR procedures after PJI-related revision arthroplasty in two studies and that this study found an infection control rate of 56 %, a DAIR procedure should still be a treatment option for PJI after revision arthroplasty. Nonetheless, the aforementioned studies show the same trend as reported in this study.

A limitation of this study is reflected by the retrospective design. The number of patients included in this study was relatively low, which was caused by the scarcity of PJI cases requiring DAIR. Logically, especially the number of patients with DAIR after revision surgery was low. Moreover, misclassification bias and risk factors that were present but not measured should also be accounted for. Heterogeneity of the groups can cause bias.

Our results show that even though the infection control rate may decrease after prior PJI-related revision arthroplasty, a subsequently performed DAIR procedure can retain the prosthesis in about 60 % of the patients. These findings should be confirmed prospectively in a larger group of patients. We recommend performing a prospective multicenter evaluation of DAIR treatment to give a conclusive answer. Moreover, for patients with an infection after primary arthroplasty, we found no difference in infection control rate between early postoperative and acute hematogenous infections.

## Supplement

10.5194/jbji-6-91-2021-supplement The supplement related to this article is available online at: https://doi.org/10.5194/jbji-6-91-2021-supplement.

## Data Availability

Raw data were generated at the University Medical Center Utrecht. Derived data supporting the findings of this study are available from the corresponding author, F. Ruben H. A. Nurmohamed, on request.

## Code availability

The data underlying the results presented in the study are available in the supporting SPSS information file.
