# Supplementary material for: One-year infection control rates of a DAIR (debridement, antibiotics and implant retention) procedure after primary and prosthetic-joint-infection-related revision arthroplasty – a retrospective cohort study
Source: J Bone Jt Infect. 2021 Jan 27;6(4):91–7. doi: 10.5194/jbji-6-91-2021 (PMC8129908; doi:10.5194/jbji-6-91-2021)
Supplement: The supplement related to this article is available online at: https://doi.org/10.5194/jbji-6-91-2021-supplement. [file jbji-6-91-supplement.zip › jbji-6-91-2021-supplement-title-page.pdf]

Supplement of J. Bone Joint Infect., 6, 91–97, 2021
https://doi.org/10.5194/jbji-6-91-2021-supplement
© Author(s) 2021. This work is distributed under
the Creative Commons Attribution 4.0 License.

Supplement of

One-year infection control rates of a DAIR (debridement, antibiotics and implant retention) procedure after primary and prosthetic-joint-infection-related revision arthroplasty – a retrospective cohort study

F. Ruben H. A. Nurmohamed et al.

Correspondence to: F. Ruben H. A. Nurmohamed (f.r.h.a.nurmohamed-2@umcutrecht.nl)

- jbji-6-91-2021-supplement-title-page.pdf
- Supplement file pathogens.sav
- Supplement file patient and infection characteristics.sav

The copyright of individual parts of the supplement might differ from the CC BY 4.0 License.
